# Molecular Imaging of Microglial Activation in Amyotrophic Lateral Sclerosis

**DOI:** 10.1371/journal.pone.0052941

**Published:** 2012-12-31

**Authors:** Philippe Corcia, Clovis Tauber, Johnnie Vercoullie, Nicolas Arlicot, Caroline Prunier, Julien Praline, Guillaume Nicolas, Yann Venel, Caroline Hommet, Jean-Louis Baulieu, Jean-Philippe Cottier, Catherine Roussel, Mickael Kassiou, Denis Guilloteau, Maria-Joao Ribeiro

**Affiliations:** 1 ALS Center, Department of Neurology, CHRU Bretonneau, Tours, France; 2 Université François Rabelais de Tours, Tours, France; 3 Inserm U 930, Tours, France; 4 Nuclear Medicine Department, CHRU Tours, France; 5 ALS Center, Department of Neurology, CHRU Angers, Angers, France; 6 CIC-IT 806 Ultrasons et Radiopharmaceutiques, Tours, France; 7 School of Chemistry, University of Sydney, New South Wales, Australia; 8 Brain and Mind Research Institute, Sydney, New South Wales, Australia; 9 Discipline of Med Rad Sci, Univ Sydney, New South, Wales, Australia; University of New South Wales, Australia

## Abstract

There is growing evidence of activated microglia and inflammatory processes in the cerebral cortex in amyotrophic lateral sclerosis (ALS). Activated microglia is characterized by increased expression of the 18 kDa translocator protein (TSPO) in the brain and may be a useful biomarker of inflammation. In this study, we evaluated neuroinflammation in ALS patients using a radioligand of TSPO, ^18^F-DPA-714. Ten patients with probable or definite ALS (all right-handed, without dementia, and untreated by riluzole or other medication that might bias the binding on the TSPO), were enrolled prospectively and eight healthy controls matched for age underwent a PET study. Comparison of the distribution volume ratios between both groups were performed using a Mann-Whitney’s test. Significant increase of distribution of volume ratios values corresponding to microglial activation was found in the ALS sample in primary motor, supplementary motor and temporal cortex (*p* = 0.009, *p* = 0.001 and *p* = 0.004, respectively). These results suggested that the cortical uptake of ^18^F-DPA-714 was increased in ALS patients during the “time of diagnosis” phase of the disease. This finding might improve our understanding of the pathophysiology of ALS and might be a surrogate marker of efficacy of treatment on microglial activation.

## Introduction

Amyotrophic Lateral Sclerosis (ALS) is the most frequent motor neuron disorder in adults. This condition is characterized by degeneration of both upper and lower motor neurons in bulbar and spinal territories and results in fasciculation, weakness and atrophy associated with hyperreflexia and spasticity. Progression is always fatal, leading to death from respiratory failure after a median of evolution of 36 months from onset [1], [2]. Five to ten percent of cases are familial, with dominant inheritance of the pathological trait in almost all cases [3]. The list of genes linked to familial ALS is currently growing dramatically, from the description of the first mutations in the superoxide dismutase gene (SOD1) in 1993 to the last description of the C9orf72 gene that appears to be one of the most important genetic factors in frequency in Western Europe ([3]. The relationship between ALS and genetic factors is continuously being strengthened by such findings [3], [4]. In the remaining sporadic ALS cases the pathophysiology remains unresolved. Several pathways have been proposed, with mitochondrial dysfunction, protein aggregate formation, excitotoxicity, axonal transport malfunction, mutant-derived oxidative damage, lack of growth factors and inflammation all purported to play a role in the motor neuron death process [5].

Neuroinflammation has been the focus of numerous studies using several approaches. These have mainly been based on the presence of both activated microglia, the macrophages of brain parenchyma, and infiltrates of lymphocytes in the areas of motor neuron loss in the motor cortex, brainstem, corticospinal tract and anterior horn of the spinal cord [6], [7], [8], [9]. Several explanations have been advanced to explain their role: decrease in the release of neurotrophic factors, excessive in the secretion of neurotoxic factors and modulation of glutamatergic receptor expression [7]. Assessment of microglial activation can be performed in vivo in ALS patients through neuroimaging of the 18 kDa translocator protein (TSPO) using selective TSPO radioligands which allow the exploration of microglia in neurological disorders. TSPO, formerly named peripheral benzodiazepine receptor (PBR), is part of a multimeric “protein complex” associated with the outer mitochondrial membrane of many cells [10]. TSPO is present in peripheral tissues and also in glia cells (astrocytes and microglia) when activated [11]. TSPO is involved in many processes such as apoptosis, regulation of cellular proliferation, immunomodulation and steroidogenesis [12]. TSPO may therefore be a useful biomarker of inflammation, since it is highly expressed in phagocytic inflammatory cells, such as activated microglia in the brain and macrophages in peripheral tissue. A large number of positron emission tomography (PET) or single photon emission computerized tomography (SPECT) radioligands selective for the TSPO have been developed, of which ^11^C-PK11195 was the first TSPO radioligand to be evaluated [13], [14]. However, the 20-minute radioactive half-life of ^11^C-PK11195 is a serious barrier to increasing the accessibility of biomarkers for routine clinical purposes, as the use of these markers is limited to centres with an on-site cyclotron. Consequently, fluorine-18-labelled ligands appear to be the best alternative, as the 110-minute half-life of fluorine-18 allows the centralised production and loco-regional delivery. In this way, several groups have evaluated rat models of neuroinflammation using a TSPO radioligand, the ^18^F-DPA-714, and they have concluded that it provides accurate quantitative information of the density of TSPO after cerebral ischemia, herpes encephalitis and gliomas [15], [16], [17], [18]. In a previously study, our group have demonstrated that the regional distribution of ^18^F-DPA-714 was in good agreement with other PET studies using TSPO radioligands in the human brain [16].

The aim of this study was to evaluate microglial activation in ALS concomitantly with the diagnosis of this disease.

## Methods

### Radiosynthesis

N,N-diethyl-2-(2-(4-(2-fluoroethoxy)phenyl)-5,7-dimethylpyrazolo[1,5-α]pyrimidin-3-yl)acetamide (DPA-714) was labelled with fluorine-18 at its 2-fluoroethyl moiety following nucleophilic substitution of the corresponding tosylate analogue according to slight modifications of previously reported procedures [15]. The formulation of ^18^F-DPA-714, leading to a sterile injectable solution of isotonic sodium chloride with ethanol in a mass percentage of less than 8% for a total injected volume ranging from 3 to 5 mL, was in accordance with the European Pharmacopoeia.

### Subjects

The study was approved by the Medical Bioethics Committee of Tours-Centre Region and conducted according to French legislation and European directives.

Ten successive ALS patients and eight healthy controls matched for age gave informed written consent before inclusion in the study. All were right-handed. All ALS patients fulfilled the criteria of probable or definite ALS according to the Airlie House meeting and did not suffer from dementia according the Mini Mental State Examination (MMSE) and the Frontal Assessment Battery (FAB) test scores of at least 28/30 and 18/18, respectively [19], [20], [21]. Disease severity was estimated using the revised ALS Functioning Rating Scale (ALSFRS-R), the disease specific rating scale graded from 48 (normal function) to 0 [22]. None of them were treated by riluzole at the time of PET evaluation in order to avoid any interaction between TSPO and this drug. Disease duration at the time of investigation was calculated from the occurrence of first symptom onset to the date of PET imaging (mean 11.7 months; range 5–27 months).

The control sample included 7 women and 1 man. Mean age at inclusion was 56.5 years (range 51.2–67.6 years). They were all illness free (brain, heart and psychiatric diseases) on the basis of screening by medical history and physical examination.

None of the participants had contraindications to MRI, and risk of pregnancy was excluded in women of child bearing age with a negative serum pregnancy test obtained in the 7 days before the PET study. All participants had not taken any drugs that might interfere with ^18^F-DPA-714 binding for at least one month before the study.

### Imaging Data Acquisition

Brain MRI was performed for all subjects using a 1.5 Tesla imager (GE Healthcare). T2-weighted images were used to reveal hypothetical brain lesions for each subject. In addition, T1-weighted SPGR acquisition with inversion-recovery was performed to allow 3D reconstruction of MR images.

Subjects were examined using a Dual Gemini (Philips Medical Systems), a whole-body hybrid PET-CT scanner. The Dual Gemini is an open PET-CT system that combines a helical dual slice CT and a 3D PET scanner equipped with its own transmission source. Acquisition data were processed with the standard package delivered with the system (PET view software-Philips Medical Systems). For cerebral studies, a low dose CT helical scan was performed first (scan field of 600 mm, increments of 5 mm, slice thickness 3.2 mm, pitch of 1.5, 0.75 second per rotation, matrix 512×512, 120 kV, 80 mAs).

All cerebral PET examinations were acquired in list mode over 90 minutes following i.v. injection of 264±59 MBq (range 193 to 304 MBq) of ^18^F-DPA-714.

### PET Data Analysis

PET sinograms were corrected for tissue attenuation, decay, scatter and randoms radiation and were reconstructed using a 3D iterative RAMLA algorithm into voxels of 2×2×2 mm^3^ and a spatial resolution of approximately 5-mm at the center of the field of view. For each subject, a pseudo-perfusion image was created as the sum of the first 6 min of emission data. This image, which reflects the initial flow-dependent activity, has been shown to be well correlated with perfusion [23]. To avoid possible bias caused by the distribution of ^18^F-DPA-714 in late frames, the transformation that brings each dynamic scan into Talairach space was estimated by co-registering the corresponding pseudo-perfusion image with a PET ^18^F-FDG template. The non-rigid registration was performed with PMOD® 3.2, using normalized mutual information.

The ^18^F-DPA-714 binding data were quantified using Logan graphical analysis, as it has few stringent requirements compared to other reference-based semi-quantitative methods. This model requires the definition of a region from which to draw the normal kinetic behavior that does not contain specific ligand binding, However, microglia cells are distributed throughout the entire brain, and in a disease such as ALS no clear reference region may exist [23], [24]. Cluster analysis was therefore performed on the dynamic PET scan of each subject to determine a suitable reference region [24], [25], [26]. The aim of this method is to classify tissue time activity curves (TAC) according to their shape and magnitude into uniform classes that are mutually exclusive. An in-house implementation of the cluster analysis method of Wong et al. developed in MATLAB was used to segment the voxels into 10 clusters [23]. The central TAC of each class was compared to a normalized mean TAC created from healthy patients, and the reference TAC was selected using a Chi-squared test with significance level of *p*<0.05. Parametric images of distribution volume ratio (DVR) of ^18^F-DPA-714 were generated.

PET images were analyzed with the regions of interest (ROI) for primary motor, supplementary motor, temporal, occipital, cerebellum and frontal cortex and thalamus and pons as defined in the MNI-AAL atlas.

### Statistical Analysis

Subjects DVR values from left and right regions were combined and averaged in the calculation of the DVR for each anatomical region. Mann-Whitney test was used to compare the DVR values obtained in the two groups. A significance level of *p* = 0.05 was used for all inferences. Coefficient of correlation was evaluated between ^18^F-DPA-714 DVR and the age of the 18 subjects studied. Coefficients of correlation were also calculated between ^18^F-DPA-714 DVR and the disease duration and ALSFR-R.

## Results

The clinical characteristics of ALS patients are summarized in Table 1. No adverse or subjective effects were observed after injection of an average of 264±59 MBq ^18^F-DPA-714 in any of the eighteen subjects studied. There was no significant difference between both groups concerning age at inclusion (*p* = 0.28).

**Table 1:** Personal and clinical characteristics of ALS patients.

Patient	Gender	Site of onset	Age at onset (yrs)	ALSFRS-R	MMSE	FAB	Disease duration at inclusion (months)
ALS1	F	Bulbar	69.5	45	28	17	5.6
ALS2	M	Bulbar	56.7	46	30	18	2.9
ALS3	M	Bulbar	58.5	42	30	18	26.9
ALS4	M	Upper Limbs	65.5	46	30	18	14.4
ALS5	M	Bulbar	55.5	40	30	18	9.9
ALS6	M	Bulbar	53.9	42	30	18	9.7
ALS7	F	Lower limbs	62.0	36	30	18	22.9
ALS8	F	Lower limbs	67.0	43	30	18	5.1
ALS9	M	Bulbar	45.6	38	30	18	13.8
ALS10	F	Lower limbs	51.9	44	30	18	5.4

**Figure 1:**
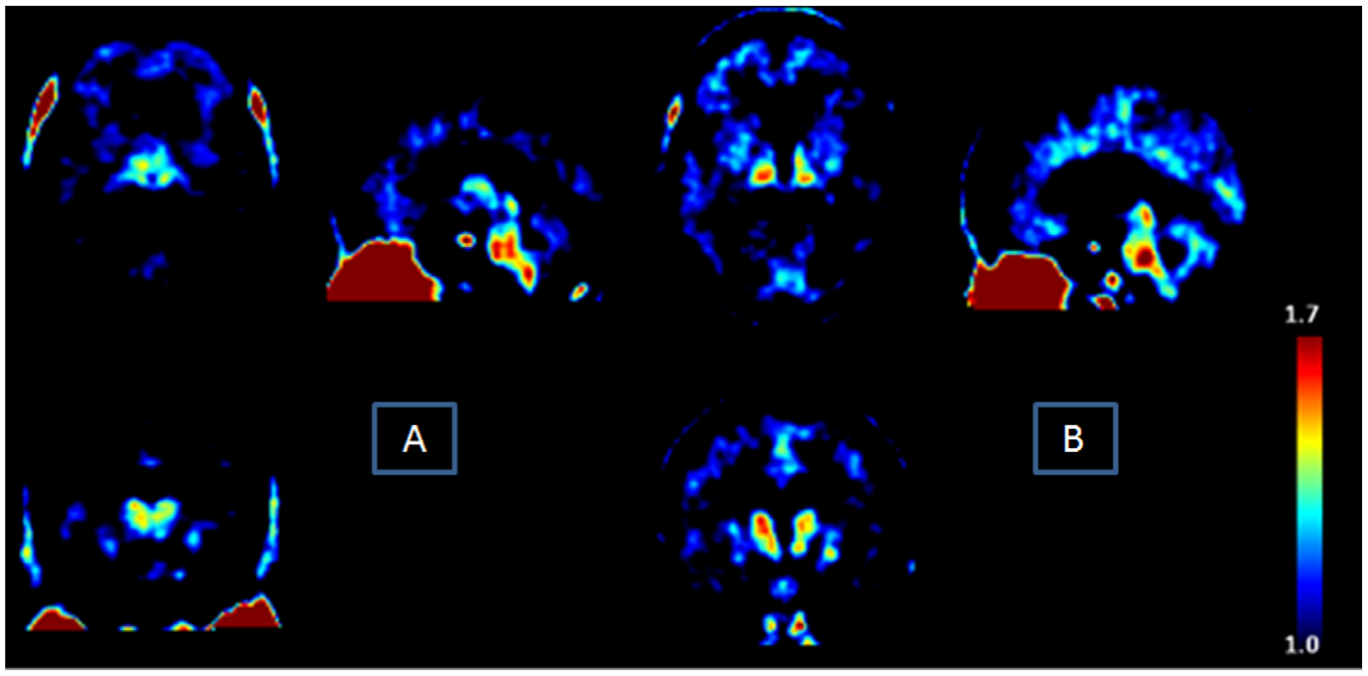
Parametric (DVR) 18F-DPA-714 images (axial, coronal and sagittal slices) obtained for a healthy volunteer and an ALS patient, respectively. Note the significant but normal 18F-DPA-714 uptake in nasal epithelium (a tissue rich in TSPO).

Figure 1 shows DVR ^18^F-DPA-714 images obtained for a control (A) and an ALS patient (B). Variable binding of ^18^F-DPA-714 was seen in all regions in all patients and controls, with overlapping of the ranges of the two groups.

**Figure 2:**
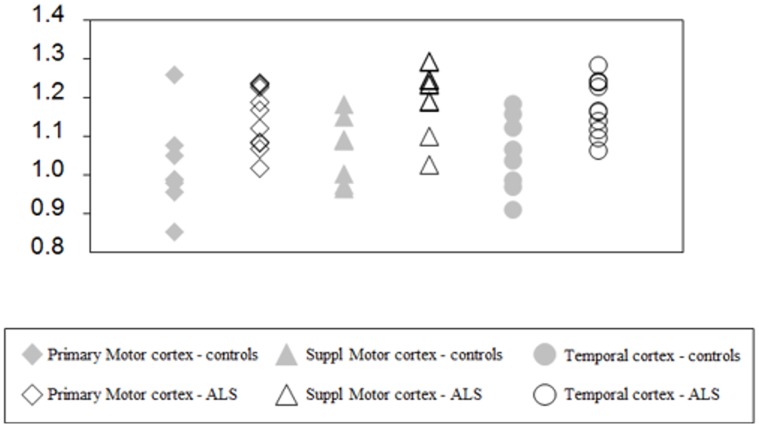
Individual DVR values for the two motor cortex regions and the temporal cortex obtained for all subjects. These are the regions where statistically significant differences were observed between the two groups studied.

^18^F-DPA-714 DVR values for all the ROI studied are summarized in Table 2. DVR values were higher in the ALS patients than in the control group and the difference reached significance in primary motor, supplementary motor and temporal ROIs (Mann-Whitney test, *p* = 0.009, *p* = 0.001 and *p* = 0.004, respectively) (figure 2 and Table 2). There was no difference in ^18^F-DPA-714 uptake in the occipital and frontal cortex, thalamus, cerebellum and pons between the two groups. No correlations were found between DVR values and age, disease duration or ALSFR-R.

**Table 2:** Individual and mean±SD ^18^F-DPA-714 DVR values obtained for the 8 healthy controls and the 10 ALS patients and results of Mann-Whitney test.

Subject	Primary Motor	SupplementaryMotor	Temporal	Occipital	Frontal	Thalamus	Cerebellum	Pons
Controls								
HC1	1.26	1.15	1.18	1.16	1.41	1.57	1.09	1.81
HC2	0.99	1.09	0.97	0.98	1.07	1.22	1.02	1.61
HC3	1.08	1.18	1.16	1.03	1.17	1.48	1.11	1.76
HC4	0.96	1.00	0.98	1.04	1.03	1.31	0.95	1.52
HC5	0.98	0.96	1.04	1.04	0.99	1.25	1.11	1.56
HC6	0.85	0.97	0.91	0.99	0.85	1.18	0.97	1.36
HC7	0.99	1.09	1.06	1.00	0.97	1.40	1.16	1.84
HC8	1.05	1.09	1.12	1.01	1.18	1.43	1.09	1.75
**Mean**	**1.02**	**1.07**	**1.05**	**1.03**	**1.08**	**1.35**	**1.06**	**1.65**
**SD**	**0.12**	**0.08**	**0.10**	**0.06**	**0.17**	**0.14**	**0.08**	**0.16**
ALS patients								
Bulbar onset								
ALS1	1.12	1.25	1.16	0.96	1.24	1.38	0.99	1.65
ALS2	1.24	1.24	1.23	1.11	1.12	1.53	1.09	1.65
ALS3	1.17	1.19	1.12	1.03	1.02	1.30	0.98	1.58
ALS5	1.23	1.24	1.24	1.12	1.18	1.52	1.11	1.81
ALS6	1.23	1.24	1.28	1.10	1.36	1.55	1.18	1.64
ALS9	1.19	1.29	1.17	1.11	1.11	1.54	1.27	1.68
Spinal onset								
ALS4	1.08	1.23	1.24	1.09	1.13	1.58	1.14	1.63
ALS7	1.02	1.03	1.06	1.00	1.04	1.20	1.05	1.35
ALS8	1.07	1.10	1.09	1.03	1.08	1.41	1.14	1.52
ALS10	1.09	1.29	1.24	1.08	1.16	1.56	1.13	1.76
**Mean**	**1.14**	**1.21**	**1.18**	**1.06**	**1.14**	**1.46**	**1.11**	**1.63**
**SD**	**0.08**	**0.08**	**0.07**	**0.05**	**0.10**	**0.13**	**0.09**	**0.12**
Wilcoxon-test (*p* value)	**0.009**	**0.001**	**0.004**	**0.155**	**0.197**	**0.0563**	**0.096**	**0.376**

DVR: Distribution of volume ratios -SD: Standard deviation.

Mann-Whitney-test: comparison between HC subjects (n = 8) and ALS patients (n = 10).

Since there was a predominance of bulbar ALS in our sample, we compared that sub-group with our healthy controls. We observed, for bulbar onset patients, an increase of ^18^F-DPA-714 binding for the three same cortical regions, primary (*p* = 0.002) and supplementary (*p*<0.001) motor, and temporal (*p* = 0.002) cortex. For the other regions, occipital cortex, thalamus, cerebellum and pons, there was no difference between the control group and the 6 ALS patients with a bulbar onset form.

## Discussion

Evidence of microgliosis in ALS has mainly been obtained from post-mortem studies and was focused on the final stages of the disease. Analysis of microglial activation at earlier stages and during the progression of the disease was technically limited until the development of neuroimaging ligands, the first being ^11^C-PK11195. PET imaging has made possible in vivo studies of microglial activation in ALS patients [25]. However, ^11^C-PK11195 had several drawbacks due to its relatively low brain plasma protein binding, a poor signal-to-noise ratio, high lipophilicity and a short physical half-live of 20 min [16]. Additional radioligands labeled with fluorine-18 have been developed, including ^18^F-DPA-714, a TSPO agonist that displays higher binding (Ki = 7.0 nM) than ^11^C-PK11195 (Ki = 9.3 nM), with a higher signal-to-noise ratio [15], [16].

In our study, we compared the ^18^F-DPA-714 binding in several cerebral regions between ALS patients and a group of healthy controls. The areas with the highest microglial activation in ALS patients were the motor cortex and more specifically the supplementary motor area. As expected, occipital and cerebellar regions were spared. Both findings were in accordance with pathology findings in ALS. This allowed us to consider that the binding of microglia fitted with the areas involved in ALS degeneration.

We did not evidence any correlation between DVR values (whatever the ROI considered) and the clinical severity of the disease estimated with the ALSFR-R score. This finds is in agreement with Turner et al and probably due to the fact that this functional scale is more accurate to evidence lower motor neuron involvement [25].

Although our sample was small, with a predominance of bulbar ALS, which is a limitation of this study, we did not find significant microglial activation in the pons. This result was not in agreement with the findings obtained by Turner et al, who highlighted significant activation in this area. We did not separate the right pons from the left pons in this study, owing to the difficulty of delineating two ROIs in such a small anatomic region [25]. However, we have founded an increase binding of ^18^F-DPA-714 in motor cortical areas in patients with bulbar form.

Otherwise, our control sample included seven women on a group of 8 subjects, whether there is a male predominance in ALS. This is no doubt a limitation of this study but women (recruited from the environment of the patients) were more volunteered to participate in this study. We did not find any reference in the literature about the existence of a difference in cerebral TSPO density distribution between men and women, but we cannot exclude its existence. In fact, TSPO is involved in many processes such as apoptosis, regulation of cellular proliferation, immunomodulation as also the production of steroid hormones and consequently TSPO density could be different in men and women.

One of the main finds of this study is that microglial activation was not restricted to motor areas. Although this result was already mentioned by Turner et al, the “extra-motor” brain areas with increased PET signal were surprisingly not the same when comparing the 2 studies. In fact, Turner et al evidenced a higher uptake of ^11^C-PK11195 in cerebral brain regions of ALS patients including pons, thalamus, and frontal cortex. In the present, ^18^F-DPA-714 binding was significantly enhanced in temporal cortex, a brain region without significant microglial activation in the previous ^11^C-PK11195 study. Even if this discrepancy must be approached cautiously regarding the sample size (10 ALS patients in both studies; a predominant bulbar onset form in our study while Turner and colleagues evaluated mainly spinal forms), and the considerable individual variability of the measured PET signal, we could hypothesized that this discrepancy points changes in microglial activation as the disease evolves. Indeed, since the delay between first symptoms and PET was significantly shorter in our study than for the study using ^11^C-PK11195 (Mann-Whitney-test, *p* = 0.006), we are able to postulate that we performed microglial PET examination at an early stage of the disease. Thus, thalamic microglial activation shown in Turner et al might be consecutive to a delayed neuroinflammatory process associated with secondary neuronal degeneration in brain areas remote from the motor cortex. Moreover, the two studies did not use the same TSPO radioligand and, as we previously described, ^11^C-PK11195 and ^18^F-DPA-714 present different characteristics.

The difference in the uptake of ^18^F-DPA-714 between ALS and controls reached one of the two highest levels of significance in medial temporal areas. Evidence of the spread of the neurodegenerative process has been suggested since the beginning of the 20th century with the description of diffuse involvement of the cerebral cortex and of the basal ganglia in ALS patients. This was corroborated by the description of diffuse involvement of the cerebral cortex and the basal ganglia in ALS patients [27].

The involvement of the temporal lobe emphasized in this study might correspond to previous neuroimaging, neuropsychological and neuropathology findings. First, neuroimaging studies emphasized atrophy of the gray matter of the temporal lobe and of white matter adjacent to this area in voxel-based morphometric analysis [28], [29]. Second, neuropsychological assessment of verbal fluency also supported this assumption. Verbal fluency might be split into two components, i.e. phonemic fluency related to the frontal lobe and semantic fluency under the control of the temporal lobe. There is a significant decrease in semantic fluency in ALS compared to controls [30]. Neuropathology studies have emphasized the presence of TDP-43 inclusions, the major disease protein in ALS, in around 40% of the temporal lobe in ALS patients [31]. Finally, a post-mortem study revealed the presence of neuronal loss and gliosis in 12.6% of ALS patients without dementia [32]. Since there was a significant association between bulbar onset and temporal lesions and inclusions, we could not exclude the possibility that the significant binding only in the temporal region was the result of the predominance of bulbar ALS in this small sample [32]. On the other hand, this result might indicate that this binding of ^18^F-DPA-714, biomarker of TSPO, in the temporal lobe at the early stages of the disease was an early marker of cognitive disorders probably preceding the occurrence of neuropsychological impairment, considered to affect 10 to 50% of ALS patients [33]. We did not perform a focused neuropsychological assessment in our population and limited our exploration to routine tests in order to exclude dementia and major frontal dysfunction. This assumption needs to be confirmed by additional studies combining PET imaging and a more comprehensive neuropsychological evaluation of semantic language fluency and episodic verbal memory in particular.

The concept of a continuum between ALS and fronto temporal lobe dementia (FLTD) has been put forward in views of the pathology and genetic overlap between the two disorders [34]. There is currently a growing body of literature supporting a continuum between ALS and FLTD. This is based on the occurrence of both conditions within the same family and of motor neuron signs and fronto temporal cognitive disorders, on neuropathology and genetic findings in the same patient [35], [36]. Our study strongly indicated that this overlap might exist since both conditions might evolve concomitantly in the onset of the disease, according to these findings. A prospective clinical, neuropsychological and imaging analysis of ALS and FTLD populations in order to assess cognitive impairment and motor damage in ALS and FLTD, respectively, would complement this study.

A recent publication demonstrated the existence of different binder’s populations for the ^11^C-PBR28, for which about 10% of the population appeared to be non-binders [37]. Owen and colleagues evidenced three types of binding pattern: high-affinity binders (about 50%), low-affinity binders (about 20%) and mixed-affinity binders (about 30%) and extended this finding to other PET TSPO radioligands, including the carbon 11-radiolabelled derivative of ^18^F-DPA-714, namely DPA-713 [38]. Although, and this is a limitation of our study, we have not directly identified subjects with low or high affinity for TSPO. In fact, it is likely that ^18^F-DPA-714 is also sensitive to this inter-individual variable affinity state.

In conclusion, our results strongly suggested an increase expression of TSPO on PET imaging using ^18^F-DPA-714 in untreated ALS patients. This increase of ^18^F-DPA-714 cortical binding is independent of the disease duration.

The ability to assess microglial activation in vivo might improve our understanding of mechanisms leading from neuroinflammation to neurodegenerative disorders and allow effective monitoring of treatment. Finally, the presence of extra motor microglial activation might corroborate theories suggesting that ALS is a prototypic motor neuron condition whose pathology is not limited to motor neurons.
